# Effects of Feed Contaminant Deoxynivalenol on Plasma Cytokines and mRNA Expression of Immune Genes in the Intestine of Broiler Chickens

**DOI:** 10.1371/journal.pone.0071492

**Published:** 2013-08-20

**Authors:** Khaled Ghareeb, Wageha A. Awad, Chimidtseren Soodoi, Soleman Sasgary, Alois Strasser, Josef Böhm

**Affiliations:** 1 Department for Farm Animals and Veterinary Public Health, Institute of Animal Nutrition and Functional Plant Compounds, University of Veterinary Medicine, Vienna, Austria; 2 Department of Animal Hygiene, Behaviour and Management, Faculty of Veterinary Medicine, South Valley University, Qena, Egypt; 3 Clinic for Avian, Reptile and Fish Medicine, Department for Farm Animals and Veterinary Public Health, University of Veterinary Medicine, Vienna, Austria; 4 Department of Biomedical Sciences, Institute of Physiology, Pathophysiology and Biophysics, University of Veterinary Medicine, Vienna, Austria; University of San Francisco, United States of America

## Abstract

An experiment was conducted to investigate the individual and combined effects of dietary deoxynivalenol (DON) and a microbial feed additive on plasma cytokine level and on the expression of immune relevant genes in jejunal tissues of broilers. A total of 40 broiler chicks were obtained from a commercial hatchery and divided randomly into four groups (10 birds per group). Birds were reared in battery cages from one day old for 5 weeks. The dietary groups were 1) control birds fed basal diet; 2) DON group fed basal diet contaminated with 10 mg DON/ kg feed; 3) DON + Mycofix group fed basal diet contaminated with 10 mg DON/ kg feed and supplemented with a commercial feed additive, Mycofix® Select (MS) (2.5 kg/ton of feed); 4) Mycofix group fed basal diet supplemented with MS (2.5 kg/ton of feed). At 35 days, the plasma levels of tumor necrosis factor alpha (TNF-α) and interleukin 8 (IL-8) were quantified by ELISA test kits. Furthermore, the mRNA expression of TNF-α, IL-8, IL-1β, interferon gamma (IFNγ), transforming growth factor beta receptor I (TGFBR1) and nuclear factor kappa-light-chain-enhancer of activated B cells 1 (NF-κβ1) in jejunum were quantified by qRT-PCR. The results showed that the plasma TNF-α decreased in response to DON, while in combination with MS, the effect of DON was reduced. DON down-regulated the relative gene expression of IL-1β, TGFBR1 and IFN-γ, and addition of MS to the DON contaminated diet compensates these effects on IL-1β, TGFBR1 but not for IFN-γ. Furthermore, supplementation of MS to either DON contaminated or control diet up-regulated the mRNA expression of NF-κβ1. In conclusion, DON has the potential to provoke and modulate immunological reactions of broilers and subsequently could increase their susceptibility to disease. The additive seemed to have almost as much of an effect as DON, albeit on different genes.

## Introduction

Deoxynivalenol (DON), or vomitoxin, is produced by *Fusarium graminearum (Gibberella zea)* and *F. culmorum* [1] and considered the most common contaminant in poultry feedstuffs. It has negative effects on growth, feed consumption and may induce intestinal alterations, neurological and reproductive problems [2]. However, immune impairment is considered the most important outcome of DON mycotoxicosis [3]. It was shown that DON has both immunostimulatory and immunosuppressive effects according to concentration, time and duration of exposure [2]. DON can be immunotoxic at low dietary concentrations even if there is no alteration of the productivity traits [3], [4], [5]. Unfortunately, limited information is available regarding the immunotoxicity of DON in poultry. In broiler chickens, DON was shown to suppress the vaccination response to infectious bronchitis virus (IBV) [6] and to Newcastle disease virus (NDV) [6], [7]. Recently, DON was shown to suppress the antibody response to infectious bronchitis vaccine (IBV) in broiler chickens [8], [9]. Furthermore, it was shown that the dietary inclusion of DON in diets of laying hens resulted in a reduction of white blood cell number and total lymphocyte number [10].

The ability of DON to affect cytokines is important because this can lead to dysregulation of immune functions. In domestic pigs, lower IL-1β and IL-8 expression occurred in blood and ileal tissue after feeding of low doses of DON [11]. Similarly, in broiler chickens, splenic mRNA expression of IFN-γ was down-regulated as a result of chronic feeding of diets naturally contaminated with DON and *Fusarium* mycotoxins [12].

Cytokines are secreted proteins that regulate the nature of immune responses by affecting growth, differentiation, and activation of cells. They are involved in almost all stages of immunity and inflammation, and cytokine production is induced by a variety of stimuli such as viral, bacterial or parasitic infection, cancer, inflammation, or the interaction between T cells and antigens. The biological actions of cytokines are produced when they act as ligands and bind to their high-affinity receptors. Cytokines can act by an autocrine manner, affecting the cell that releases the cytokine, or by paracrine manner, affecting nearby cells, or by an endocrine manner, affecting distant cells after their distribution via the blood circulation [13], [14]. There is a lack of information about the impacts of DON on the plasma level of cytokines.

Furthermore, the gastrointestinal tract (GIT) is considered as important barrier against toxins and contaminants [15] which has significant physical, chemical, immunological and microbiological characteristics. Intestines are large immune organs and have a broad capability for innate and acquired immune reactions against various antigens [16]. A variety of soluble mediators have been shown to participate in the gastrointestinal inflammatory process, including the pro-inflammatory interleukins [17]. Thus, it becomes important to investigate the effects of DON on the relative expression levels of immune genes, including cytokines and transcription factors, in the intestines of broiler chickens.

Microbial feed additives are frequently used to reduce the negative impacts of DON. It has been shown that Mycofix can reduce the negative impact of DON on vaccinal immune response to IBV, stress index and blood lymphocyte DNA [8], [18]. Therefore, the current study was conducted to assess the effects of five weeks dietary exposure to DON and a microbial feed additive on the plasma levels of TNF-α and IL-8 and on the expression of TNF-α, IL-8, IL-1β, TGFBR1, IFNγ, TGFBR1 and NF-κβ1 in jejunum of broiler chickens. TNF-α and IL-8 were selected for investigation in the plasma because both are involved in the systemic inflammation, activation of the acute phase reaction, and mediation of inflammatory responses in order to overcome the infectious antigens. In addition, TNF-α and IL-8 are known targets of NF-κB, which is modulated in cases of DON toxicity as a result of activation of mitogen activated protein kinases [11]. To our knowledge, this is the first *in vivo* study which observes these parameters in broiler chickens.

## Materials and Methods

### Ethics statement

The animal experiments were discussed and approved by the institutional ethics committee of the University of Veterinary Medicine and Austrian Federal Ministry for Science and Research under the license number GZ-68.205/0032-II/10b/2010. All husbandry practices and euthanasia were performed with full consideration of animal welfare.

### Experimental design, birds and diets

A total of forty 1d male broiler chicks (Ross 308) were obtained from a commercial hatchery and divided randomly into 4 groups (10 birds per group). Birds of each group received one of the following dietary treatments; 1) control birds fed basal diet; 2) DON group fed basal diet contaminated with 10 mg DON/ kg feed; 3) DON + Mycofix group fed basal diet contaminated with 10 mg DON/ kg feed and supplemented with 2.5 kg of a commercially microbial feed additive, Mycofix^®^ Select (MS), (Biomin GmbH, Herzogenburg, Austria) per ton of feed; 4) Mycofix group fed basal diet supplemented with 2.5 kg of a commercially microbial feed additive, Mycofix^®^ Select, (Biomin GmbH, Herzogenburg, Austria) per ton of feed. The birds were fed the starter feed from 1–13 days old and grower feed from 14–35 days old (Table 1). Water and feed were available *ad libitum*. The climatic conditions and lighting program were computer-operated and followed the commercial recommendations. Environmental temperature in the first week of life was 35°C and reduced to 25°C until the end of the experiment. During the first week, 22 h of light was provided with a reduction to 20 h afterward. Representative feed samples were taken at the beginning of the starter and grower periods and were analyzed for nutrient and mycotoxin content. Levels of deoxynivalenol, acetyldeoxynivalenol, zearalenone, nivalenol, and fusarenon-X were determined in the diets using an HPLC technique [19].

**Table 1:** Composition and analysis of the experimental diet (%).

Ingredient	Starter	Grower
**Corn**	55.0	62.0
**Soya high protein**	29.0	23.80
**Rapeseed oil**	1.00	2.00
**Soybean roasted**	6.91	5.53
**Calcium carbonate**	2.03	1.62
**Monocalcium phosphate**	1.89	1.71
**Palm oil**	1.88	1.50
**Pumpkin seed expeller** 1	0.72	0.58
**Sodium chloride**	0.48	0.38
**L-Lysine**	0.34	0.27
**Methionine**	0.24	0.20
**Vitamin Premix** 2	0.16	0.13
**Magnesium phosphate**	0.13	0.10
**L-Threonine**	0.12	0.10
**Trace element premix** 3	0.10	0.08
**Deoxynivalenol** 4 **(mg/kg)**		
**Mycofix Select** 5		
**Calculated composition**		
**DM**	89	89
**CP**	21.5	18.8
**ME (MJ/kg)**	12.6	13
**Crude fiber**	2.6	2.6
**Crude fat**	6.5	7.2
**Lys**	1.44	1.21
**Met**	0.56	0.48
**Ca (g/kg)**	1.2	1
**P (g/kg)**	0.86	0.72
**Na (g/kg)**	0.2	0.16
**Mg (g/kg)**	0.18	0.17
**Analyzed composition**		
**CP**	23.9	20.9
**Crude fat**	7.0	7.6
**Ca (g/kg)**	14.9	11.2
**P (g/kg)**	8.7	8.7
**Na (g/kg)**	2.85	1.98
**Mg (g/kg)**	3.2	2.5

1 Pumpkin seed expeller is a byproduct of oil manufacture, obtained by pressing of pumpkin seeds, Cucurbita maxima Duch, moschata (Duch) Poir., Cucurbita pepo L., and other species of Cucurbita.

2 Produced by MIAVIT GmbH & Co. KG, Essen (Oldb.), Germany. Each kilogram of vitamin premix contains vitamin A, 200,000 IU; vitamin D3, 80,000 IU; vitamin E, 1,600 mg; vitamin K3, 34 mg; vitamin C, 1,300 mg; vitamin B1, 35 mg; vitamin B2, 135 mg; vitamin B6, 100 mg; vitamin B12, 670 μg; nicotinic acid, 1,340 mg; calcium pantothenic acid, 235 mg; choline chloride, 8,400 mg; folic acid, 34 mg; biotin, 3,350 μg; and methionine, 30 g.

3 Produced by MIAVIT GmbH & Co. KG, Essen (Oldb.), Germany. Each kilogram of trace element premix contains calcium, 196 g; phosphorous, 64 g; sodium, 30 g; magnesium, 6 g; copper, 400 mg; zinc, 1,200 mg; iron, 2,000 mg; manganese, 1,200 mg; cobalt, 20 mg; iodine, 40 mg; selenium, 8 mg.

4 Ten milligrams of deoxynivalenol/kg were added to constitute the DON group.

5 Two and half kg of Mycofix® Select (Biomin GmbH, Herzogenburg, Austria)/ton of diet were added to constitute the Mycofix® group. This plus 10 mg DON/kg of diet constitutes DON+Mycofix group.

### Measurement of plasma cytokine level

At 35 days old blood samples from all experimental birds were collected in heparinized tubes, and plasma of each bird was separated by centrifugation at 1000g for 15 min for determination of TNF-α and IL-8 levels. The concentrations for tumor necrosis factor alpha (TNF-α) and interleukin 8 (IL-8) were quantified in plasma using commercially available ELISA test kits (Antibodies-online.com, Aachen, Germany).

### Quantification of mRNA of immune genes by quantitative (q) RT-PCR Sample Extraction

Intestinal tissue samples from mid-jejunum (5 cm from Meckel's diverticulum) from all birds (10 birds/group) were removed immediately after slaughter (stunning followed by bleeding). The epithelial layer was collected in Petri dishes on ice by scraping with a sterile scalpel and immersed in the RNA stabilizing agent, RNA-later (Qiagen, Hilden, Germany) and frozen at −80°C. After thawing, 30 mg of the tissue was used to extract total RNA with a commercial extraction kit (RNeasy Protect Mini Kit, Qiagen, Hilden, Germany). The RNA amount in extracts was determined fluorospectrometrically with an EvaGreen RNA determination kit (Invitrogen, Karlsruhe, Germany). For cDNA synthesis, total RNA was diluted to 0.2 μg/μL in diethylpyrocarbonate-treated water. Reverse transcription into cDNA was performed using a High Capacity cDNA Archive Kit (Applied Biosystems, Darmstadt, Germany) according to manufacturer's instructions included in the kit. The cDNA was diluted 1 ∶64 and stored at −20°C until further analysis.

### Quantitative Real-Time PCR (qRT-PCR)

A commercial master mix (OneStep RT-PCR Kit, Qiagen, Hilden, Germany) with the addition of EvaGreen as fluorescent agent (0.08 nmol/L, final concentration) was used for amplification of GAPDH, TNFα, IL-8, IL-1β, TGFBR1, IFNγ, TGFBR1 and NF-κβ1. The oligonucleotide primers used for PCR amplification were synthesized (Table 2).

**Table 2:** Oligonucleotide primers used for real-time PCR (RT-PCR).

Gene	Real-time PCR, sense (5′-3′)	Antisense (5′-3′)
**GAPDH**	CCATCACAGCCACACAGAAGAC	TGGACGCTGGGATGATGTT
**IFNγ**	GTGAAGAAGGTGAAAGATATCATGGA	GCTTTGCGCTGGATTCTCA
**IL-1β**	CAGCCTCAGCGAAGAGACCTT	CACTGTGGTGTGCTCAGAATCC
**IL-8**	CTGGCCCTCCTCCTGGTT	GCAGCTCATTCCCCATCTTTAC
**NF-κB1**	GCAACTATGTTGGACCTGCAAA	ACCCACCAAGCTGTGAGCAT
**TNFα**	CCCCTACCCTGTCCCACAA	TGAGTACTGCGGAGGGTTCAT
**TGFBR1**	ACCAGGTCCTACTCCAGGAAGAC	AAAGCAGACAGGTCCAGCAATAA

The following PCR program was employed on the ViiA™ 7 System (Applied Biosystems, Vienna, Austria) to amplify target mRNA in tissue extracts: initial denaturation for 15 min at 95°C (1 cycle); followed by 45 cycles of 30 sec 95°C and 50 sec 63°C. A dissociation curve was generated after 45 cycles in order to determine melting points of the amplified cDNA.

### Calculation of relative mRNA Expression

GAPDH was investigated as a reference gene based on expression stability. The expression of genes of interest (GOIs) was normalized using the housekeeping gene GAPDH. Data were analyzed using the efficiency corrected Delta-Delta-Ct method [20]. The mRNA expression of TNFα, IL-8, IL-1β, TGFBR1, IFNγ, and NF-κβ1 were evaluated in jejunum.

### Statistical Analysis

The statistical program SPSS (version 17; SPSS GmbH, SPSS Inc., Munich, Germany) was used for data analysis. The Kolmogorov-Smirnov test was used to test the normal distribution of the data. An ANOVA was performed between the 4 groups, followed by Duncan test to find the significance between dietary treatments. The probability values of 0.05 (P≤0.05) were considered significant.

## Results

### Mycotoxin contents of diets

The dietary concentration of DON in the starter control feed was 506±80 μg/kg, and the level of the zearalenone was under the limits of detection. In the DON-contaminated starter feed the concentration was 10,509±80 μg/kg, and other mycotoxins were under the limits of detection. In the DON non-contaminated control grower diet, DON concentration was 611±95 μg/kg and in the DON contaminated grower diet; DON was 10,614±95 μg/kg. Other mycotoxins were under the limits of detection.

### Plasma cytokines production

In plasma, TNF-α was significantly down-regulated in broiler chickens receiving DON (P<0.05) compared to the control group (Table 3). Moreover, addition of MS counteracted the effect of DON on plasma TNF-α and the value was comparable to that of control birds. Differently, IL-8 did not significantly alter (P>0.05) for all the broiler chickens receiving DON, counteracting agent or DON and the counteracting agent compared to the control group.

**Table 3:** Effects of DON and a microbial feed additive on the plasma cytokines.

Parameters	Control group	DON group	DON+Mycofix	Mycofix group	P value
TNF α (µg/ml))	116^a^±7	53^b^±5	105^a^±20	115^a^±22	0.033
IL-8 (µg/ml)	2.83±0.21	2.24±0.45	3.22±1.01	3.39±0.67	0.619

### Relative mRNA expression of immune genes

**Figure 1:**
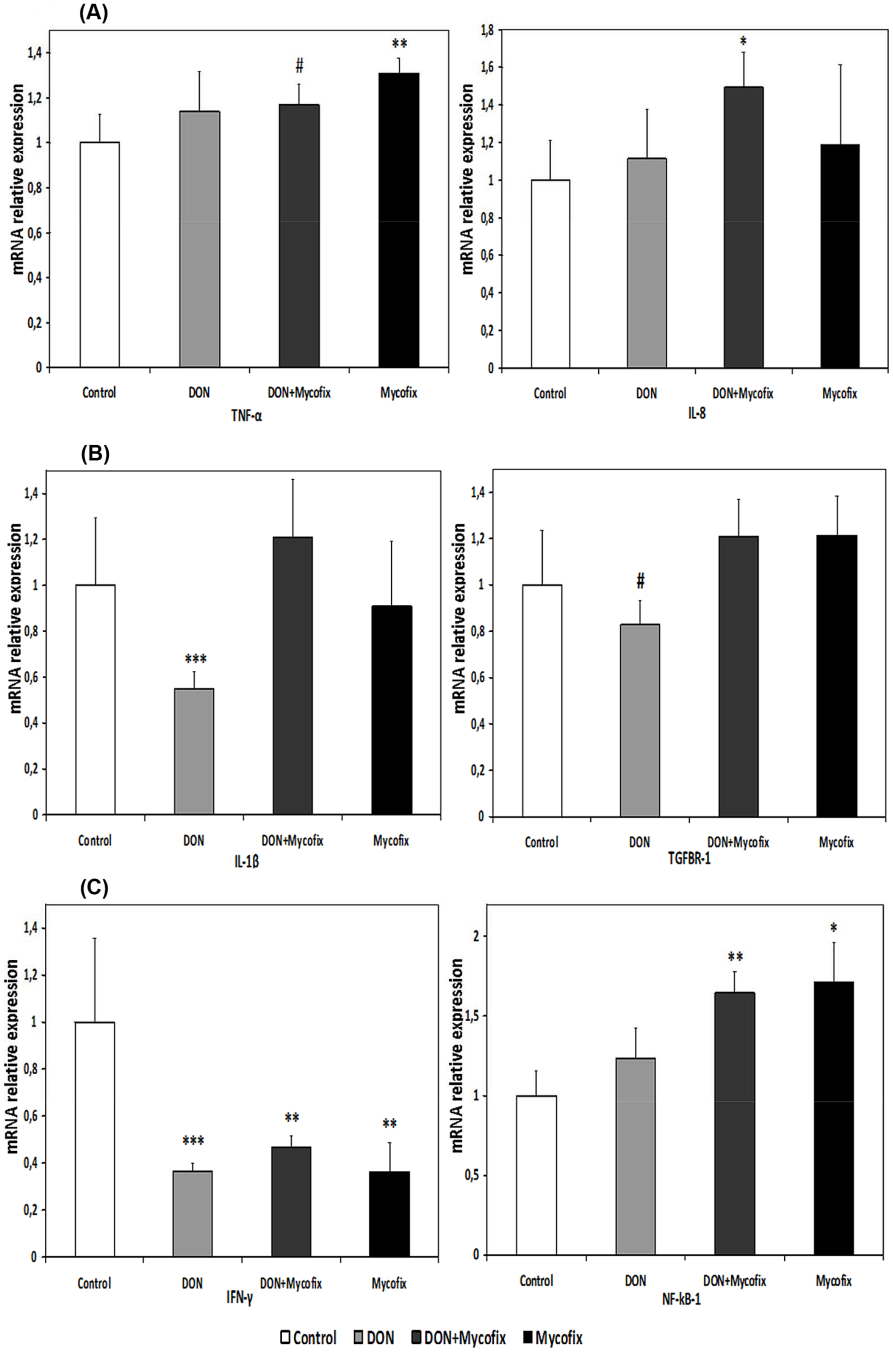
The relative mRNA expression of immune genes in the jejunal tissues of broiler chickens at 35 days after feeding of experimental diets was quantified by qRT-PCR. The mRNA expression of controls and birds fed with DON or DON+Mycofix or Mycofix was compared for (A) TNF-α and IL-8, (B) IL-1β and TGFBR1, as well as for (C) IFN-γ and NF-κβ1. The relative gene expression for each gene was calculated relative to the gene expression of the control group. Bars show the means and standard errors of means (SEM). GAPDH was chosen as a reference gene having the same relative expression mean in the control as in the treated groups. Values are the means of 5 birds. The difference in relative expression of immune gene between birds fed experimental diets and birds fed control basal diet was assessed by Student's t-test and comparisons were considered significantly different at P<0.05 (*), P<0.01 (**), and *P*<0.001 (***) or as a trend for a difference at *P*<0.1 (^#^).

DON down-regulated (P<0.001) the mRNA relative expression of IL-1β in jejunal tissue. However, feed supplementation with the counteracting agent was efficient to increase the mRNA relative expression of IL-1β to values that were comparable to control birds (Figure 1B). Furthermore, DON feeding resulted in a trend for down-regulation (P<0.1) of TGFBR-1 in the jejunal tissue of broilers (Figure 1B). However, Mycofix addition to either DON contaminated diet or basal control diet did not produce a significant change on the relative gene expression of TGFBR-1 and IL-1ß (Figure 1B). Moreover, the relative mRNA expression of IL-8 was up-regulated (P<0.05) when Mycofix was added to DON contaminated diet (P<0.05; Figure 1A). Interferon-gamma (IFN-γ) relative gene expression was significantly down-regulated (P<0.001) due to DON feeding, as well as when Mycofix was added to either DON contaminated feed or to basal control feed (Figure 1C).

Mycofix supplementation either with or without DON significantly (P<0.05) up-regulates the expression of NF-κB1 (Figure 1C). Furthermore, the relative mRNA expression of TNF-α also up-regulated (P<0.05) due to Mycofix supplementation and tended to be up-regulated (P<0.1) when MS was added to DON contaminated feed (Figure 1A).

## Discussion

The impairment of immune function is the most important toxic effect of DON and may result in either immune-stimulation or immune-suppression depending on the dose, time and duration of exposure. The results of this study represent new information regarding the impacts of deoxynivalenol oral exposure on plasma cytokines and expression of immune genes, including the genes that regulate the production of pro-inflammatory cytokines in the gastrointestinal tract of broiler chickens.

DON is known to either suppress or stimulate immunological parameters such as inhibition of lymphocyte proliferation with a concomitant elevation of immunoglobulin and cytokine concentrations *in vitro* or *in vivo*, even at permissible levels [3], [4], [21], [22]. The phenomenon that protein synthesis inhibitors up-regulate cytokine gene expression and secretion is called “superinduction” and may be due to inhibition of particularly labile translational repressor proteins [23], [24], [25]. However, it should not be ignored that immunotoxicity studies have focused mainly on the mouse model with only few investigations in domestic animals. Therefore, the current experiment was conducted to investigate the impacts of DON and a microbial feed additive on plasma levels of cytokine TNF-α and interleukin 8 (IL-8) and on the relative gene expression of TNF-α, IL-8, NF-κB1, IFN-γ, IL-1β, and TGFBR1 in the jejunum of broilers.

Interleukins are a group of cytokines that are important components of the immune system. They play a physiological role in inflammation and a pathological role in systemic inflammatory states, and any imbalance in cytokine production and dysregulation of a cytokine process could result in various pathological disorders [26]. In the present study, plasma level of IL-8 showed less change due to the addition of either DON or Mycofix Select but these changes were not statistically significant. Oral DON decreased the production of IL-8 which mediates the inflammatory immune response, suggesting that DON ingestion could impair immune function and elevate susceptibility to disease. On the other hand, addition of Mycofix Select to either DON contaminated diet or to basal control diet resulted in a smaller increase of IL-8 production, indicating that this feed additive improved the immune response of chickens, probably due to its immune stimulating properties.

The current state of knowledge indicates that DON can have marked immunological effects in chickens. Feeding of 10 mg DON/kg feed to broilers decreased the plasma concentration of TNF-α in the present study. TNF-α is a cytokine involved in systemic inflammation and stimulation of the acute phase reaction. It is produced chiefly by activated macrophages (M1), although it can be produced by a variety of cells such as CD4+ lymphocytes, NK cells, and neurons. Large amounts of TNF-α are released in response to lipopolysaccharide, other bacterial products, and other cytokines such as interleukin (IL)-1, IL-2, IFN-γ, IFN-α, Granulocyte Macrophage Colony Stimulating factor (GM-CSF), the Transforming Growth Factor (TGF)-ß [27].

The primary role of TNF is in the regulation of immune cells. It is possible that DON exposure resulting in decreased levels of TNF-α contributes to impairment of immune function in poultry and increased susceptibility to infectious diseases when birds are fed DON for long periods. However, this hypothesis needs further studies to be confirmed. In contrast, feeding of DON contaminated diet (2.9 mg/kg feed) did not affect the TNF α level in the plasma of pigs [28], and pigs that received both LPS and DON induced TNF-α compared with controls. The difference between chickens and pigs in the term of sensitivity to DON toxicity, the dose, and period of exposure could explain the difference between both studies regarding TNF-α level in the plasma. However, in comparison to pigs that received LPS alone, DON and LPS pigs had lower levels of plasma TNF α, indicating that DON reduced plasma TNF α in the presence of LPS [28]. In the current study, the addition of a microbial feed additive counteracted the reducing level of TNF-α in the plasma of broiler fed DON, suggesting that this feed supplement was beneficial for the reduction of DON effects on plasma cytokines. Furthermore, birds receiving DON and/ or Mycofix Select appeared clinically healthy without any clinical signs during the whole experimental period and their daily body weight gain did not differ between dietary treatments.

It was shown that DON is able to phosphorylate Iκβ in human Caco-2 cells [29], resulting in release of the transcription factor (Nuclear Factor kappa β, NF-κβ) from its inhibitor Iκβ, which then allows NF-κβ to translocate into nucleus and activate the transcription of specific genes [30]. Deoxynivalenol was found to increase the binding activity of NF-κβ *in vitro* [31] through inhibition of resynthesis of IκBα, a member of Iκß family.

Nuclear Factor kappa β (NF-kβ) is a transcription factor that is activated by various intra- and extra-cellular stimuli such as cytokines, oxidant-free radicals, and bacterial or viral products. NF-κß stimulates the expression of genes involved in a wide variety of biological functions. It is located normally and in a steady state in the cytoplasm in association with the inhibitory Iκß protein in an inactive form [32]. The activated transcription factor (NF-κβ ) then translocates to the nucleus of the cell [33], [34], [35], [36], [37] and switches on gene expression for cytokines (i.e. activates the transcription of pro-inflammatory genes) and subsequent release of proinflammatory mediators [38] such as tumor necrosis factor-α (TNF-α) and interleukins [39].

In this study, addition of DON caused a significant reduction of mRNA levels of some genes (IL-1β, TGFBR1, IFN-γ) but caused no changes in others (TNF-α, IL-8, NF-κβ). This is in contrast to DON action in the usual mouse model but fits with DON action in pigs. In mice, it was found that the levels of mRNA of IL-1ß, IL-6, and TNF-α, IFN-γ, IL-4 and IL-10 were increased after a single oral dose of 5 and 25 mg/kg body weight [40]. The capability of DON to affect the cytokine gene expression is important because it can indicate the modulation of immune functions. It was shown that up-regulation of the relative gene expression of NF-κβ 1, TNF-α and IL-8 in case of DON toxicity due to the decrease of the protein synthesis in the intestinal cells [23], [24], [25]. However, in domestic pigs, lower IL-1β, IL-8 and TNF-α expressions occurred in blood and ileal tissue after feeding of low doses of DON [11], suggesting the down-regulation of immune-related transcription factors and pro-inflammatory immune factors of pigs fed DON. Similarly, feeding of low (3.44 mg/ kg feed), medium (6.30 to 10.68 mg/kg feed), and high (16.97 mg/kg feed) deoxynivalenol for 3 weeks experiment did not alter IL-8 expression in the cecal tonsils of broilers [12].

In contrast, IL-1β and IFN-γ was down-regulated after oral DON exposure in the present experiment. This result is in agreement with the previous report of Cheng *et al*. [41],which found that in pigs treated with DON for 6 weeks, a decrease in the expression of IFN-γ, IL-1β was observed. Similarly, in broiler chickens, splenic mRNA expression of IFN-γ was down-regulated as a result of chronic feeding of naturally contaminated diets with DON and other *Fusarium* mycotoxin contaminated diet [42]. In another studies, IFN-γ gene expression was up-regulated in the cecal tonsils of chickens fed *Furarium* mycotoxin challenged with coccidia [43]. In this context, it becomes evident that DON has a modulating effect on the innate immune response. Moreover, the decline of IFN-γ expression may impair the anti-virus ability of the host. Cell-mediated and humoral immune response network are also interfered by a decrease of IL-1β level. Cytokines play an important role in the network of immune responses and account for communication, activation, maturation and differentiation among immune cells. Since dietary DON can modify gene expression of cytokines, it may impair disease resistance in poultry.

Furthermore, DON had a similar down-regulating effect on transforming growth factor beta receptor I (TGFBR1), which is a cytokine protein secreted by many cell types, including macrophages. TGFBR1 plays a crucial role in the regulation of the cell cycle and regulates a variety of different cellular developmental processes including growth, differentiation, proliferation, and cell death. TGF-β is believed to be important in regulation of the immune system by regulatory T cell and in the differentiation of both regulatory T cell and Th17 cells. TGF-β appears to block the activation of lymphocytes and monocyte derived phagocytes. In the current study, DON down-regulated this cytokine in the jejunal tissue of broilers. In addition, a microbial feed additive when added to DON contaminated diet counteracted the immune modulation of DON on TGFBR1, and IL-1β. However, it has no counteracting effects on down-regulation of the relative mRNA expression of IFN-γ caused by DON. The ability of Mycofix Select to reverse the effects of DON for some genes but not others could be related to the dose of the feed additive added to DON contaminated diet. In this study, 2.5 kg Mycofix Select per ton of diet was used, which could not be enough to reverse all the effects of of 10 mg DON/ kg feed. Interestingly, Mycofix in and of itself increased the levels of several mRNAs that were not affected by DON (.TNF-α, IL-8 and NF-kB), This indicates that addition of feed additive Mycofix Select stimulates the immune response genes, suggesting that it has an immune stimulating property. This information may be important as Mycofix Select is used commercially as antidote for mycotoxin.

In conclusion, DON reduced the level of plasma TNF-α and down-regulated the relative mRNA expression of IFN-γ, IL-1β, and TGFBR1, which indicates the ability of DON to inhibit the protein synthesis. However, mRNA relative expression of IL-8, NF-κB and TNF-α were up-regulated, suggesting that DON could have an effect on the innate immune response which can impair the resistance of chickens to infectious diseases and consequently increase the susceptibility of the host to infection. Furthermore, a microbial feed additive has the ability to modulate the DON effects on plasma level of TNF-α and on the relative expression of mRNA of IL-1β and TGFBR1 in the intestinal epithelium of broiler chickens.
