# Correction: Effects of feed contaminant deoxynivalenol on plasma cytokines and mRNA expression of immune genes in the intestine of broiler chickens

**DOI:** 10.1371/journal.pone.0100051

**Published:** 2014-06-06

**Authors:** The PLOS ONE Staff

There is an error in the affiliation for one of the co-first authors. The correct affiliation for Wageha A. Awad is #2 Department of Animal Hygiene, Behaviour and Management, Faculty of Veterinary Medicine, South Valley University, Qena, Egypt, and #3 Clinic for Avian, Reptile and Fish Medicine, Department for Farm Animals and Veterinary Public Health, University of Veterinary Medicine, Vienna, Austria.
